# Challenges in the use of nanostructures as carriers of nucleic acids in clinical practice

**DOI:** 10.31744/einstein_journal/2022RB5898

**Published:** 2022-02-03

**Authors:** Carolina Capobiango Romano Quintão, Luiz Sérgio de Almeida Camargo, Humberto de Mello Brandão, Naiara Zoccal Saraiva, Michele Munk

**Affiliations:** 1 Empresa Brasileira de Pesquisa Agropecuária Juiz de Fora MG Brazil Empresa Brasileira de Pesquisa Agropecuária, Juiz de Fora, MG, Brazil.; 2 Universidade Federal de Juiz de Fora Juiz de Fora MG Brazil Universidade Federal de Juiz de Fora, Juiz de Fora, MG, Brazil.

**Keywords:** Nucleic acids, Nanostructures, Embryonic structures, Nanotubes, carbon, Animals, genetically modified

## Abstract

The delivery of nucleic acids to cells is considered a crucial step for the success of genetic modifications aimed at therapeutic purposes or production of genetically modified animals. In this context, nanotechnology is one of the most promising fields of science, with the potential to solve several existing problems. Nanostructures have desirable characteristics to be used as carriers, such as nanometric size, large surface area, cell internalization capacity, prolonged and controlled release, among others. Genetically modified animals can contribute to the production of biopharmaceuticals, through the expression of high-associated-value molecules. The production of these animals, also known as biofactories, further enhances Brazilian agribusiness, since it allows adding value to the final product, and favors the integration between the agricultural market and the pharmaceutical sector. However, there is a growing concern about the safety and possible harmful effects of nanostructures, since data on the safe use of these materials are still insufficient. The objective of this review was to address aspects of the use of nanostructures, mainly carbon nanotubes as nucleic acid carriers, aiming at the production of genetically modified animals, with the certainty that progress in this field of knowledge depends on more information on the mechanisms of interaction between nanostructures, cells and embryos, as well as on its toxicity.

## INTRODUCTION

The study of nanotechnology is recent. Many researchers in the field consider that the interest in this technology has grown since 1959, after Richard Feynman, an American physicist, stating it would be possible to build structures of nanometric sizes from the manipulation of atoms.^(1)^

Thus, nanotechnology can be understood as a set of techniques used to manipulate matter at the atomic and molecular scale, to form structures with size between 1nm and 100nm.^(2)^ At this scale, materials start to present physical-chemical properties different from those at the micro or macro scale, mainly related to electrical conductivity, elasticity, and greater mechanical resistance, among others.^(3)^

Currently, nanotechnology is considered a priority area for the advancement of technological innovation and the economic and social development of countries.^(4)^

In Brazil, after investments of approximately €140 million by the Ministry of Science, Technology, Innovation, and Communication (MCTIC - *Ministério da Ciência, Tecnologia, Inovações e Comunicações*) nanotechnology can be considered one of the strategic sectors for the government. According to a recent survey, the country showed 13% annual growth in scientific publications on nanotechnology in the period between 2000 and 2018, with the best Brazilian universities leading the way.^(5)^

In the pharmaceutical industry, the use of nanostructures in the system known as Drug Delivery Nanosystems offers advantages when compared to conventional systems, with emphasis on the protection of the drug in the body, the maintenance of the concentration at plasma levels, and the reduction of side effects from high doses, thus increasing therapeutic efficacy.^(6)^ In this context, nanotechnology emerges as an alternative to overcome the limitations of conventional systems.

Regarding the use of nanostructures in the production of genetically modified animals, previous studies have shown nanoparticles can carry exogenous genes into cells and embryos.^(7)^ However, with the promising advances in this area, there is also growing concern about the potential risks associated with these techniques, since information about their possible impacts on human health and the environment is insufficient.

The objective of this review was to present aspects involved in the use of nanostructures, with a focus on carbon nanotubes (CNT) as carriers of nucleic acids for the production of genetically modified animals, as well as to address aspects related to the toxicity and safe use of this new technology.

## INTRACELLULAR TRANSPORT OF NUCLEIC ACIDS

The knowledge of cell transfection processes, that is, the insertion of exogenous genes inside the cells, is important for the studies of gene therapies related to human diseases, and for a better understanding of the mechanisms of gene expression regulation and the methods of production of genetically modified animals.

In this sense, several cell transfection protocols have been proposed, aiming at an efficient transport system of molecules into somatic cells and embryonic structures. Among the most used gene carriers, there are those of biological nature, such as viral vectors,^(8)^ and those of physical or chemical nature.^(9)^

Viral vectors present natural abilities to reach inside the cells and take control of the cellular machinery. However, some of the challenges to their use are the limited carrying capacity due to the size of the transgene to be transported; cytotoxicity; immunogenicity; induction of undesirable genetic modifications that may cause tumors; and difficulty of large-scale use.^(10)^

The cell transfection alternatives, involving physical and chemical agents, have a limited potential, due, among other factors, to cytotoxicity and intracellular degradability, resulting in a low rate of transfected cells.^(11)^

Regarding the techniques employed for the production of genetically modified animals, the most often used are microinjection of DNA into the pronucleus,^(12)^ sperm-mediated DNA transfer,^(13)^ and nuclear transfer with genetically modified somatic cells.^(14)^ However, these techniques are laborious and have limitations, such as low success rates, high embryonic mortality, and high equipment costs.

To be used as carriers, the materials must have three main characteristics: to package and protect the target molecule from intracellular degradation; to gain access to the intended intracellular compartment; and to release the molecule into the cell in the appropriate spatiotemporal condition.^(15)^

On the other hand, when the entry of exogenous molecules into cells occurs by other methods, such as endocytosis, the process becomes less dependent on the properties of these carriers, allowing the entry of virtually any material that has submicrometric size dispersed in suspension. In some cases, the carriers also exhibit fusogenic potential, *i.e*., the ability to fuse to the membrane of the target cell.^(15)^

Nanomaterials can act as intracellular carriers, with the ability to access the cytoplasm of eukaryotic cells, due to their ability to penetrate the membrane, either by damage to the structure or endocytosis,^(7)^ the latter being the most common process of internalization. Moreover, to transfect embryonic structures, the molecules carrying nucleic acids must be able to penetrate the *zona pellucida*, one of the barriers to transfection methods used traditionally.^(7)^

Endocytosis is influenced by physicochemical properties and interactions between the nanocarrier and the target cell surface. Some characteristics, such as shape, size, surface charge, and type of carrier material directly influence the process.^(16)^ Indeed, it is known that in the case of site-directed delivery to cells, nanostructures can be functionalized with molecules that interact with specific receptors on the cell surface, and that the same nanocarrier can be taken up by different cell pathways, since this process is cell-dependent.^(16)^

Generally, after internalization, the nanocarrier is enveloped by the endocytic vesicle, which fuses to the early endosome, then to the late endosome, and finally accumulates in the lysosome, where it may be degraded, since the lysosomal pH is altered to values close to 5.0.^(16)^ This may be a limiting factor to the application of nanostructures for gene transfection,^(16)^ since some mechanisms, such as endosomal escape, are not yet fully understood.

Another factor that must be considered in the delivery of molecules into the intracellular medium is the kinetics of transporter release, directly related to the affinity of charges between the transporter and the molecule being transported, since delayed unpacking has been reported as a bottleneck for transfection efficiency.^(17)^ Additionally, the efficiency of drug or genetic material translocation into the nucleus and other target organelles may also impact transfection success.^(18)^

Thus, research directed towards the discovery or optimization of carriers for biological molecules is important for improving cell transfection rates and obtaining genetically modified products. In production animals, such as cattle, genetic modification may result in animals that are more resistant to disease, and that produce milk with lower allergenic protein content and higher quality. One must also consider the possibility that these animals act as bioreactors, in the secretion of a large volume of proteins with high biological value, through the mammary gland.^(19)^Genetically modified animals can also contribute to research on organs for xenotransplantation,^(20)^ and as animal models for studies of diseases.^(21)^

## ASPECTS INVOLVED IN THE USE OF CARBON NANOTUBES AS CARRIERS OF NUCLEIC ACIDS FOR THE PRODUCTION OF GENETICALLY MODIFIED ANIMALS

A milestone in the development of nanotechnology was the synthesis of CNT in 1991, by Iijima.^(22)^ Carbon nanotubes is a sheet of graphene rolled up to connect its ends, forming a tube. It can be formed by a single sheet of graphene, resulting in single-walled nanotubes, or multiple sheets of graphene, forming multiwalled carbon nanotubes.^(22)^

The CNT have certain desirable characteristics for their use as carriers of DNA and RNA, such as large contact surface, stability, and ability to interact with nucleic acids through hydrophobic and electrostatic interactions.^(23)^

They can be functionalized with different chemical groups, aiming at a greater interaction with cell targets, through modifications in their biological properties, which gives CNT the ability to pass through different types of cell membranes.^(24)^

**Figure 1:**
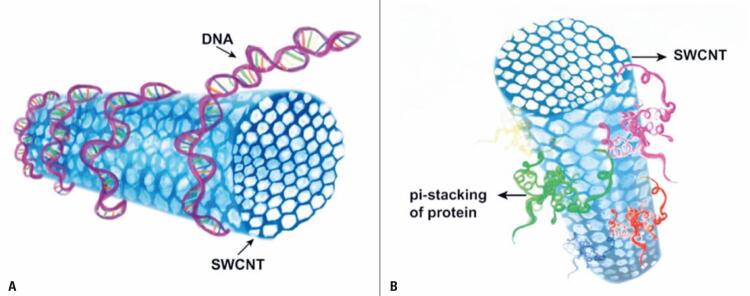
Models of molecular interactions between carbon nanotubes and biological molecules. (A) Interaction of DNA double helices on the surface of carbon nanotubes. The bases of nucleotides and proteins interact with carbon nanotubes by means of hydrophobic interactions or by van der Waals force, while the phosphate groups of the DNA molecule interact with water molecules. (B) Types of bonds existing between proteins and carbon nanotubes. Through p-p stacking, an interaction occurs between carbon nanotubes and the aromatic residues (Trp, Phe, and Tyr) of proteins, contributing to better adsorption and biocompatibility

Source: Adapted from Vardharajula S, Ali SZ, Tiwari PM, Eroğlu E, Vig K, Dennis VA, et al. Functionalized carbon nanotubes: biomedical applications. Int J Nanomedicine. 2012;7:5361-74.^(26)^

Using molecular simulation, Gao et al.,^(25)^observed DNA molecules could be encapsulated within or around CNT. The nucleotide bases and proteins interact with the CNT by means of hydrophobic interactions (Figure 1) or by van der Waals force, while the phosphate groups of the DNA molecule interact with water molecules.^(26)^ This organization occurs because DNA molecules are amphipathic, while the CNT present hydrophobic characteristics, although, when functionalized, they are able to acquire positive or negative charges, depending on the type of target molecule to be transported and its stability in aqueous suspension. Hernandez et al.,^(27)^observed the encapsulation process of the nucleic acids inside the CNT favors their protection against degradation by cell nucleases.

SWCNT: single-walled carbon nanotubes.

Additionally, the non-covalent binding of nucleic acids on the surface of CNT increases the efficiency of their release into the cell. However, transfection efficiency can be influenced by the cell type, size, and/or by the functionalization method of the CNT.^(28)^

## NANOTOXICITY OF CARBON NANOTUBES

Nanotoxicology is a branch of toxicology that aims to investigate the adverse effects of nanomaterials on human health, animal health, and the environment.

Currently, regulatory research aims to develop reliable, robust, and reproducible protocols for interlaboratory nanotoxicological testing. In this context, there is the NANoREG project, conceived by the European Union and coordinated by the Ministry of Infrastructure and Environment of the Netherlands, with the aim to promote international regulation in nanotechnology.^(29)^

**Figure 2:**
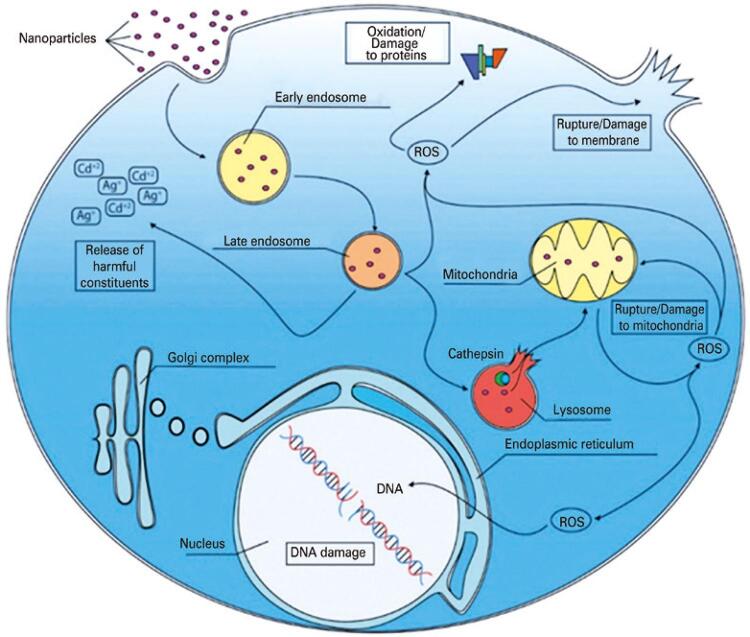
Main toxic effects triggered by nanostructures in eukaryotic cells. Nanoparticles can enter the cell mainly by endocytosis or by damage to cell membranes. Upon internalization and passage through the endosome-lysosome system, nanomaterials are normally degraded, releasing constituents that can generate reactive oxygen species. Reactive oxygen species have the potential to cause damage to the cell membrane, organelles, proteins, and nucleic acids, resulting in mutagenicity and cell death. Thus, reactive oxygen species production and DNA damage are considered the main mechanisms by which nanomaterials induce toxicity

Source: translated from Radaic A, Pugliese, GO, Campese GC, Pessine FB, Jesus MB. Como estudar interações entre nanopartículas e sistemas biológicos. Quim Nova. 2016;39(10):1236-44. Review. Figura 3, Principais efeitos tóxicos desencadeados por nanomateriais em células eucarióticas; p. 1242.^(30)^

It is interesting to note in the study of nanomaterials that their toxic potential is tied to the same characteristics that make them important for technological applications, such as nanoscale dimensions and a large surface area, since these properties increase their interaction with target cells and tissues. Some of these unwanted effects are demonstrated in figure 2.^(30)^

However, it has been shown this occurs when nanomaterials are in high concentrations in cells and tissues, possibly at sites where additional factors influence the intensity of changes in cellular homeostasis.^(31)^ Factors such as size, morphology, surface characteristics, solubility, aggregation, chemical composition, and presence of functional groups, are linked to understanding the mechanisms of toxicity of nanostructures.^(31)^

Sohaebuddin et al.,^(32)^ demonstrated that in human colon cancer cells, CNT with diameters smaller than 8nm were more cytotoxic than those with size between 20nm and 30nm, or larger than 50nm. However, in the same work, the opposite was observed for murine macrophages that were more sensitive to CNT, with diameters larger than 50nm when compared to those with diameters smaller than 8nm. Besides the effect of size, which implies an increase in surface area and reactivity, it was also observed in this study that the toxicity is cell-dependent.^(33)^

Recent research using mice as study models demonstrated that CNT modified with carboxyl groups were more biocompatible compared to amino CNT.^(34)^ This result highlights the fact that the toxicity of nanomaterials can be altered according to the presence of certain functional groups on their surface. With regard to nanotoxic effects on mammalian gametes and embryonic structures, few studies have been reported.

Carbon nanotubes seem especially suitable as delivery vectors for small molecules of DNA or RNA, but more studies are needed to evaluate their use in the production of genetically modified animals, with minimal adverse effects.

## CONCLUSION

The growing demand for new technologies capable of optimizing the intracellular delivery of drugs, with impact on the treatment of several diseases, has increased the interest of researchers in the study of nanomaterials as possible carriers of chemical or biological molecules into cells. In the same sense, animal researchers have realized these nanomaterials could also be used in the transfer of exogenous genes in the context of the production of genetically modified animals, with some advantages over existing methods. Nevertheless, with the increasing importance of this theme, there is also a growing concern about the safety in the use of these nanostructures, so that the expansion of knowledge in this area is essential to support regulatory guidelines that promote the consolidation of nanoscience in Brazil.
